# Beneficial Effect of *Taraxacum coreanum* Nakai via the Activation of LKB1-AMPK Signaling Pathway on Obesity

**DOI:** 10.1155/2021/6655599

**Published:** 2021-01-17

**Authors:** Mi-Rae Shin, Min Ju Kim, Hae-Jin Park, Jegeun Han, Seong-Soo Roh

**Affiliations:** ^1^Department of Herbology, Korean Medicine of College, Daegu Haany University, Deagu 42158, Republic of Korea; ^2^DHU Bio Convergence Testing Center, 1, Hanuidae-ro, Gyeongsan-si, Gyeongsangbuk-do 38610, Republic of Korea; ^3^WONILBIO, 41 Bioballey 2-ro, Jecheon-si, Chungcheongbuk-do 27159, Republic of Korea

## Abstract

**Objective:**

Liver kinase B (LKB) 1 and AMP-activated protein kinase (AMPK) are master regulators and sensors for energy homeostasis. AMPK is mainly activated via phosphorylation of LKB1 under energy stress. Here, we highlighted the antiobesity effect and underlying mechanism of *Taraxacum coreanum* Nakai (TCN) in connection with LKB1-AMPK signaling pathway.

**Methods:**

Male C57BL/6 mice were fed on a high-fat diet (60% kcal fat; HFD) to induce obesity. Simultaneously, they received 100 or 200 mg/kg TCN orally for 5 weeks. We measured the body weight gain and liver weight along with liver histology. Moreover, the changes of factors related to lipid metabolism and *β*-oxidation were analyzed in the liver, together with blood parameters.

**Results:**

The body weights were decreased in mice of the TCN200 group more than those of the HFD control group. Moreover, TCN supplementation lowered serum triglyceride (TG) and total cholesterol (TC) levels, whereas TCN increased HDL-cholesterol level. Liver pathological damage induced by HFD was alleviated with TCN treatment and accompanied with significant reduction in serum AST and ALT activities. In addition, TCN significantly increased the expression of p-AMPK compared with the HFD control group via the activation of LKB1/AMPK signaling pathway. Lipid synthesis gene like ACC was downregulated and factors related to *β*-oxidation such as carnitine palmitoyl transferase-1 (CPT-1) and uncoupling protein 2 (UCP-2) were upregulated through peroxisome proliferator-activated receptor (PPAR) *α* activation.

**Conclusion:**

Taken together, these data suggest that TCN treatment regulates lipid metabolism via LKB1-AMPK signaling pathway and promotes *β*-oxidation by PPAR*α*; hence, TCN may have potential remedy in the prevention and treatment of obesity.

## 1. Introduction

Obesity has continued to be a public health concern across the globe over the past decades. Obesity is primarily caused by the energy imbalance between nutrition and physical activity [1]. It is also at higher risks for developing serious health problems like dyslipidemia, fatty liver disease, hypertension, stroke, diabetes mellitus, osteoarthritis, cancers, respiratory problems, and sleep apnoea [2]. The World Health Organization (WHO) reported that more than 1.9 billion (39% of the world's adult population) in 2016 were overweight and of these over 650 million (about 13% of the world's adult population) were obese. Approximately 2.8 million deaths happened as a result of being overweight or obese [3]. Obesity is related to immune dysregulation and chronic low-grade inflammation [4]. Accordingly, that involves improving the immune response such as regular physical exercise, weight loss though caloric restriction, and use of AMP-activated protein kinase (AMPK) activators.

Reactive oxygen species (ROS) are naturally produced under many metabolic reactions, most of the production of ATP in mitochondria. Above all, ROS regulation is important for the maintenance of cellular homeostasis [5]. AMPK, which is the main sensor of cellular energy status, is activated in response to energy stress such as starvation, hypoxia, exercise, or the stimulation though drug (metformin, thiazolidinediones) and restores energy balance by promoting catabolic process that generates ATP, while inhibiting anabolic process that consumes ATP. Here, AMPK is ultimately activated by upstream kinase (liver kinase B1; LKB1) in response to stimuli like the increase of AMP/ADP ratio [6]. That is, the phosphorylation at threonine residue (Thr) 172 within the catalytic *α* subunit by LKB1 requires the process of AMPK activation. The activated AMPK affects energy-consuming pathways such as de novo lipid biosynthesis as well as energy-producing pathways such as lipid oxidation [7]. This series of processes includes the induction of phosphorylation, which means inactivation of acetyl coenzyme A carboxylase (ACC) and the decrease of malonyl-CoA. Thereby, it alleviates the inhibition of carnitine palmitoyl-transferase- (CPT-) 1 and leads to an increase in fatty acid oxidation in the liver. For that reason, AMPK is reported to regulate various metabolic processes dysregulated in classic chronic diseases such as inflammation, obesity, diabetes, and cancer [5]. AMPK is also considered as a promising target for therapeutic and pharmaceutical studies on disorders such as obesity, diabetes, and metabolic syndrome, where the body energy homeostasis is imbalanced [8].

*Taraxacum coreanum* Nakai (TCN), which is called a white dandelion in Korea and Japan, has been used as a traditional therapeutic agent for various inflammatory diseases such as gastritis, gastric ulcer, and tonsillitis [9, 10]. Its extract has been reported to have protective effects against stomach, liver, and brain damage induced by ethanol, CCl_4_, and methionine and choline deficient diets [11–13]. Ultimately, bitter substances are known as stimulating the digestion, while phenolic compounds account for the antioxidative and anti-inflammatory activities [14, 15]. Besides the above-mentioned effects, TCN has been reported to have other biological activities, including antidiabetic, antifungal, and anticancer effects [10]. Compared to roots, the leaf of TCN is characterized by higher polyphenol such as chicoric acid and chlorogenic acid and flavonoids such as luteolin and quercetin [16].

To our knowledge, previous studies have never investigated the antiobesity effect of TCN. Therefore, we selected the best extraction method and evaluated its antiobesity effect and underlying mechanism in connection with LKB1-AMPK signaling pathway with 70% ethanol extract of TCN on an experimental model of obesity.

## 2. Materials and Methods

### 2.1. Chemical and Reagents

The protease inhibitor mixture, ethylenediaminetetraacetic acid (EDTA), was acquired from Wako Pure Chemical Industries, Ltd. (Osaka, Japan). Rabbit polyclonal antibody against peroxisome proliferator activated receptor (PPAR) *α*, mouse monoclonal antibodies against *β*-actin and histone, and goat polyclonal antibody against mitochondrial uncoupling protein (UCP)-2 were purchased from Santa Cruz Biotechnology, Inc. (Santa Cruz, CA, USA). Rabbit monoclonal antibody against LKB1 and phospho-LKB1 and rabbit polyclonal antibodies against acetyl-CoA carboxylase (ACC), phospho-ACC, AMPK*α*, and phospho-AMPK*α* were purchased from Cell Signaling Technology, Inc. (Danvers, MA, USA). Goat polyclonal antibody carnitine palmitoyltransferase- (CPT-) 1 was purchased from Abcam (Cambridge, UK). ECL Western Blotting Detection Reagents and nitrocellulose membranes were supplied by GE Healthcare (Buckinghamshire, UK). Goat anti-rabbit, rabbit anti-goat, and goat anti-mouse immunoglobulin G (IgG) horseradish peroxidase- (HRP-) conjugated secondary antibodies were purchased from GeneTex, Inc. (Irvine, LA, USA). All other chemicals and reagents were obtained from Sigma-Aldrich (St Louis, MO, USA). Chicoric acid was acquired from Tokyo Chemical Industry Co., Ltd. (Tokyo, Japan).

### 2.2. Preparation of Plant Extract

**Figure 1:**
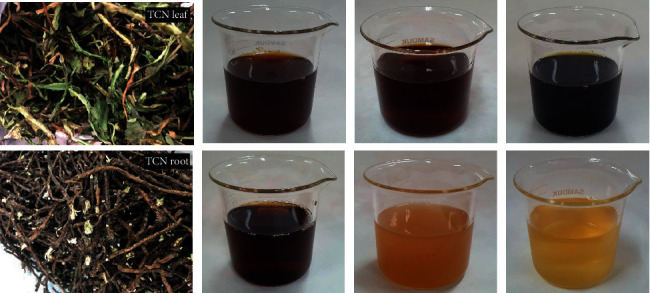
The sample extract images obtained by different extraction methods with leaf and root of TCN. (A) Water extract, (B) 30% ethanol extract, and (C) 70% ethanol extract.

The root and leaf of TCN were collected from Wonilbio (Jecheon, Korea) in 2019. These were identified by Dr Roh SS and were deposited in Daegu Haany University. Ethanolic extracts were made in the following way. Dried leaves and root parts (100 g) were soaked in 70% ethanol (1000 mL) and then left for 1 day at room temperature. Moreover, water extracts were made in the following way. Each dried leaf and root part (100 g) was prepared and extracted with 10 times of distilled water and boiled in 100°C for 2 h. The solvent was dried using a rotary evaporator following filtration with filter paper. We obtained the following yields (Table 1). And, Figure 1 shows the sample extract images obtained by different extraction methods with different parts (leaf and root) of TCN.

### 2.3. DPPH Radical Scavenging Property

Antioxidant activity determination of AT-mix was performed by the DPPH radical scavenging property according to the method of Hatano et al. [17]. In microwells, 100 *μ*L of an ethanolic solution of the sample (control: 100 *μ*L of ethanol) was added to an ethanolic solution of DPPH (60 *μ*M). After mixing gently and the reaction being carried out for 30 min at room temperature, the optical density was measured absorbance at 540 nm with a Microplate Reader (Tecan M200 PRO, Zürich, Switzerland). The antioxidant activity of each sample was expressed in terms of the IC_50_ (concentration required to inhibit DPPH radical formation by 50%) calculated from the log-dose inhibition curve. The radical scavenging activity was calculated using the following equation:

(1) $$\begin{array}{c} \text{DPPH radical scavenging property}\left(\%\right)=\left[1-\left(\frac{A_{\text{sample}}}{A_{\text{blank}}}\right)\right]\times 100. \end{array}$$

### 2.4. ABTS Radical Scavenging Property

ABTS radical scavenging activity of the different extracts was measured according to the modified method of Re et al. [18]. ABTS stock solution was dissolved in water to a 7.4 mM concentration. The ABTS radical cation (ABTS) was produced by reacting ABTS stock solution with 2.45 mM potassium persulfate and allowing the mixture to stand for 16 h at room temperature in the dark. The ABTS solution was diluted with ethanol to obtain an absorbance of 0.70 ± 0.02 at 415 nm. After adding 95 *μ*L of diluted ABTS solution (A 415 nm = 0.70 ± 0.02) to 5 *μ*L of sample, the mixture was left for 30 min at room temperature in the dark. The optical density was measured absorbance at 415 nm with a Microplate Reader. The radical scavenging property was calculated using the following equation:

(2) $$\begin{array}{c} \text{ABTS radical scavenging property}\left(\%\right)=\left[1-\left(\frac{A_{\text{sample}}}{A_{\text{blank}}}\right)\right]\times 100. \end{array}$$

### 2.5. Total Phenolic and Flavonoid Contents

The total phenolic content of TCN was quantified by mild modification from the method of Folin-Ciocalteu [19]. A 10 *μ*L TCN and distilled water 790 *μ*L were shaken well and then mixed with 50 *μ*L of Folin-Ciocalteu's reagent for 1 min. Then, 150 *μ*L of 20% sodium carbonate solution (Na_2_CO_3_) was added and the mixture was shaken for 2 h at 20°C. Finally, the absorbance of the resulting color was measured at 765 nm. The total phenolic content was expressed as mg gallic acid equivalents per gram extract. Values presented are the average of three measurements. Flavonoid was extracted and quantified by adaptation of the method of Lister et al. [20]. TCN 100 *μ*L and 1 mL diethylene glycol were mixed well. And then 1 N NaOH 10 *μ*L was added and the mixture was incubated for 1 h at 37°C. Finally, the absorbance of the resulting color was measured at 420 nm. The flavonoid content was expressed as mg naringin equivalents per gram extract. Values presented are the average of three measurements.

### 2.6. Ex Vivo Lipolysis Assay

The epididymal fat pads were removed from the male mouse (25–30 g) and placed in Hank's balanced salt solution buffer (HBSS, Invitrogen, California, USA). The removed fat pads were shredded (20 mg/piece) and prepared in 1 mL HBSS containing 1% FFA free bovine serum albumin (Sigma-Aldrich, St. Louis, USA) at 150 mg per test tube. Test drug diluted in various concentrations and isoproterenol (7.5 mM, 1.86 mg/mL), which is a positive control drug, were added to the test tube and then incubated at 37°C for 1 h. After 1 h, the supernatant was collected and the amount of glycerol spilled due to decomposition of fat was measured using a glycerol assay kit (Sigma-Aldrich, St. Louis, USA).

### 2.7. Experimental Animals and Treatment

Male healthy 5-week-old C57BL/6 mice (about 20–25 g) were purchased from DBL Co., Ltd. (Eumseong, Korea). Each mouse was kept at room temperature (23 ± 2°C) and humidity (50 ± 10%) with a 12-hour light/dark cycle. The mice were allowed free access to laboratory pellet chow and water ad libitum. After adaptation (1 week), all experimental mice except normal mice (Normal, *n* = 8) were fed with 60% high-fat diet (HFD; Diet 12492, Research Diets, Inc., New Brunswick, NJ, USA) for 5 days to adapt to a feed. Thereafter, C57BL/6 mice (*n* = 32) fed HFD were randomly divided into four groups (*n* = 8 in each group): HFD control group (Control), *Garcinia cambogia* extract group 200 mg/kg/day (GCE200), and two TCN treatment groups 100 or 200 mg/kg/day (TCN100 or TCN200). The normal and HFD control groups were given water using a stomach tube, while the drug treatment groups were orally administered GCE or TCN daily using a stomach tube for 5 weeks. *Taraxacum* (dandelion) has been used in traditional Chinese medicine and dietary application; its adverse effects are rare. Oral administration of dandelion did not lead to any toxic sign or death in mice, and LD_50_ was established to be greater than 20 g/kg body weight [21]. For that reason, this experiment was conducted by selecting 200 mg/kg body weight, a concentration frequently used in obesity experiments induced by a high-fat diet. After administration for 5 weeks, each mouse was anesthetized after fasting for 12 h. The serum was immediately separated from the blood by centrifugation and kept at −80°C until analysis.

### 2.8. Measurement of TG, TC, LDL-Cholesterol, and VLDL-Cholesterol Contents in Serum

The blood was centrifuged at 1,500 × *g* for 10 min at 4°C. Serum triglyceride (TC) and total cholesterol (TG) were conducted spectrophotometrically using commercially available kits (Wako Pure Chemical Industries, Ltd., Osaka, Japan). HDL-cholesterol was measured using a commercial kit from Asan Pharm. Co., Ltd., (Hwaseong, Korea, Cat. AM203). LDL-cholesterol was calculated though TG, TC, and HDL-cholesterol levels and VLDL-cholesterol was calculated though TG level.

(3) $$\begin{array}{c} \text{LDL}-\text{cholesterol level}\left(\text{mg}/\text{dL}\right)=\left[\text{TC}-\left(\text{HDL}-\text{cholesterol}\right)-\frac{\text{TG}}{5}\right],\\ \text{VLDL}-\text{cholesterol level}\left(\text{mg}/\text{dL}\right)=\frac{\text{TG}}{5}. \end{array}$$

### 2.9. Preparation of Cytosol and Nuclear Fractions

Protein extraction was performed according to the method of Komatsu with minor modifications [22]. Liver tissues for cytosol fraction were homogenized with ice-cold lysis buffer A (250 mL) containing 10 mM HEPES (pH 7.8), 10 mM KCl, 2 mM MgCl_2_, 1 mM DTT, 0.1 mM EDTA, 0.1 mM PMSF, and 1,250 *μ*L protease inhibitor mixture solution. The homogenate was incubated at 4°C for 20 min. And then 10% NP-40 was added and mixed well. After centrifugation (13,400 × *g* for 2 min at 4°C) using Eppendorf 5415R (Hamburg, Germany), the supernatant liquid (cytosol fraction) was separated in a new e-tube. The left pellets were washed twice by buffer A and the supernatant was discarded. Next, the pellets were suspended with lysis buffer C (20 mL) containing 50 mM HEPES (pH 7.8), 50 mM KCl, 300 mM NaCl, 1 mM DTT, 0.1 mM EDTA, 0.1 mM PMSF, 1% (v/v) glycerol, and 100 *μ*L protease inhibitor mixture solution suspended and incubated at 4°C for 30 min. After centrifugation (13,400 × *g* for 10 min at 4°C), the nuclear fraction was prepared to collect the supernatant. Both cytosol and nuclear fractions were kept at −80°C before the analysis.

### 2.10. Immunoblotting Analyses

For the estimation of PPAR*α* and histone, 12 *μ*g of nuclear fraction was electrophoresed using 10% sodium dodecylsulfate polyacrylamide gel (SDS-PAGE). Separated proteins were transferred to a nitrocellulose membrane and then incubated with primary antibodies (PPAR*α* and histone) overnight at 4°C. Then, they were incubated with anti-mouse or anti-rabbit IgG HRP-conjugated secondary antibody for 1 h 30 min at room temperature. In addition, 8 *μ*g proteins of each cytosol fraction of p-LKB1, LKB1, p-AMPK*α*, AMPK*α*, p-ACC, ACC, UCP-2, CPT-1, and *β*-actin were electrophoresed through 8–12% SDS-PAGE. Each antigen-antibody complex was detected by chemiluminescence with Sensi-Q 2000 Chemidoc (Lugen Sci Co., Ltd., Gyeonggi-do, Korea). Band densities were measured using the ATTO Densitograph Software (ATTO Corporation, Tokyo, Japan) and quantified as the ratio to *β*-actin or histone. The protein expression levels between the groups are expressed relative to those of the normal group (represented as 1). We followed the methods of Shin et al. [23] and Kim et al. [24] regarding the experimental method.

### 2.11. Oil Red O Stain in the Liver

The microscopic analysis of the effect of TCN treatment on lipid accumulation of the HFD-fed mouse liver was carried out though Oil Red O stain. The frozen liver tissue was cut into 7 *μ*m with Probe-On-Plus Slides (Thermo Fisher Scientific, Massachusetts, U.S.) and affixed to microscope slides. Sections were reacted with Oil Red O solution buffer for 7 min at 60°C. Then, they were incubated with 85% propylene glycol for 3 min. After rinsing with water, sections were stained with Harris hematoxylin for counterstaining. The stained slices were subsequently observed under an optical microscope and analyzed by the i-Solution (Innerview Co., Seongnam, Korea).

### 2.12. Statistical Analysis

Data are expressed as mean ± SEM and mean ± SD. Statistical comparisons were performed by one-way ANOVA followed by LSD test (SPSS 25.0 for Windows, SPSS Inc., U.S.A.) and values of *p* < 0.05 were considered significant.

## 3. Results

### 3.1. Comparative Evaluation of Antioxidant Efficacy In Vitro for the Selection of TCN Sample Used in This Experiment

**Figure 2:**
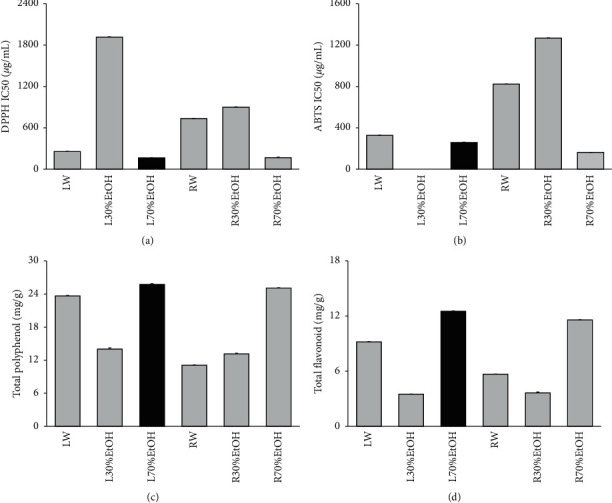
Comparative evaluation of antioxidant efficacy in vitro for the selection of TCN sample used in this experiment. (a) DPPH free radical scavenging activity, (b) ABTS free radical scavenging activity, (c) total polyphenol content, (d) total flavonoid content. LW: water extract of TCN (leaf); L30%EtOH: 30% ethanol extract of TCN (leaf); L70%EtOH: 70% ethanol extract of TCN (leaf); RW: water extract of TCN (root); R30%EtOH: 30% ethanol extract of TCN (root); R70%EtOH: 70% ethanol extract of TCN (root). Data are mean ± SEM. Each experiment was run in triplicate.

DPPH and ABTS radical scavenging activities were performed to compare antioxidant activities according to parts (leaf and root) and extraction methods (water, 30% ethanol, and 70% ethanol) of TCN. Antioxidant activity is expressed in terms of IC_50_, and IC_50_ (*μ*g/mL) represents concentration of sample providing 50% of radical scavenging activities like DPPH and ABTS assays. As shown in Figure 2, IC_50_ values of DPPH radical scavenging activity of leaf and root extracted in 70% ethanol extract were 164.06 ± 2.51 and 166.51 ± 6.3 *μ*g/mL. Also, IC_50_ value of ABTS radical scavenging activity was 257.12 ± 3.77 and 162.7 ± 0.6 *μ*g/mL. The 70% ethanol extract showed better radical scavenging than that of water extract. Subsequently, assays for the content measurement of the phenolic compound or flavonoid were performed. Total phenolic content of 70% ethanol extract (leaf) was the best among the six samples as 25.74 ± 0.17 mg GAE/g of TCN extract. The flavonoid content also had the highest content as 12.53 ± 0.03 mg naringin equivalent (NE)/g of TCN extract. Taken together, 70% ethanol extract (leaf) was most excellent in all antioxidant capabilities.

### 3.2. Ex Vivo Lipolysis Assay for the Selection of TCN Sample Used in This Experiment

**Figure 3:**
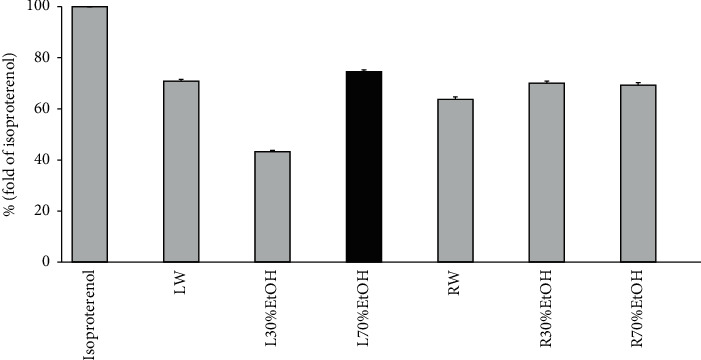
Ex vivo lipolysis assay for the selection of TCN sample used in this experiment. LW: water extract of TCN (leaf); L30%EtOH: 30% ethanol extract of TCN (leaf); L70%EtOH: 70% ethanol extract of TCN (leaf); RW: water extract of TCN (root); R30%EtOH: 30% ethanol extract of TCN (root); R70%EtOH: 70% ethanol extract of TCN (root). Data are mean ± SEM. Each experiment was run in triplicate.

The ex vivo lipolysis assay was performed to compare lipolysis effects according to parts (leaf and root) and extraction methods (water, 30% ethanol, and 70% ethanol) of TCN. As shown in Figure 3, the amount of glycerol spilled of leaf extracted using 70% ethanol extract was 74.63 ± 0.59% (fold of isoproterenol). Leaf sample extracted using 70% ethanol extract among 6 sample extracts was the most outstanding sample in lipolysis effect.

### 3.3. Body Weight Gain, Liver Weight, and Food Efficiency Ratio

Table 2 shows body weight gain, liver weight, and food efficiency ratio (FER) during the experimental periods. As shown in Table 2, mean body weights of all groups started without a significant difference between 22.27 g and 22.99 g. The normal group increased slightly in weight gain during the experimental period of 5 weeks and the level showed a significant difference compared with the HFD control group (*p* < 0.001). The GCE200 and TCN200 treated groups were significantly decreased compared with the HFD control group (GCE200; *p* < 0.001, TCN200; *p* < 0.01). The HFD consumption led to the increase of liver weight. Besides, drug treatment significantly decreased (GCE200, TCN200; *p* < 0.001, TCN100; *p* < 0.01). Moreover, the food intake and food efficiency ratio between the HFD supplied groups did not show a significant difference. As a result, TCN treatment decreased body weight gain without a significant change of FER.

### 3.4. Biochemical Analyses

**Figure 4:**
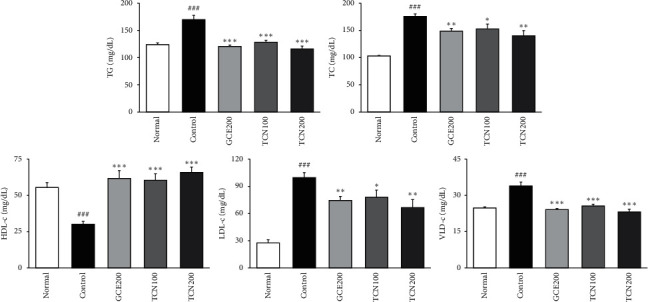
Biochemical analyses. Normal: normal mice; Control: HFD control mice; GCE200: GCE 200 mg/kg-treated and obese mice; TCN100: TCN 100 mg/kg-treated and obese mice; TCN200: TCN 200 mg/kg-treated and obese mice. Data are the mean ± SD (*n* = 8). Significance: ^###^*p* < 0.001 versus the normal mice. ^*∗*^*p* < 0.05, ^*∗∗*^*p* < 0.01, ^*∗∗∗*^*p* < 0.001 versus the HFD control mice. TG: triglyceride; TC: total cholesterol; LDL-c: low-density lipoprotein-cholesterol; HDL-c: high-density lipoprotein-cholesterol; VLDL-c: very low-density lipoprotein-cholesterol.

HFD caused the increase in serum TG, TC, HDL-cholesterol, LDL-cholesterol, and VLDL-cholesterol (*p* < 0.001, respectively). The elevated TG and VLDL-cholesterol levels were significantly reduced by all drugs compared with the HFD control group (*p* < 0.001). Moreover, the augmented TC and LDL-cholesterol levels were significantly lowered by GCE and TCN administration compared with the HFD control group (GCE200; *p* < 0.01, TCN100; *p* < 0.05, TCN200; *p* < 0.01), whereas the administration of GCE and TCN significantly elevated HDL-cholesterol level (*p* < 0.001). Particularly, TG and VLDL-cholesterol in the TCN200 group showed lower values than those of the normal group (Figure 4).

### 3.5. Histological Changes

**Figure 5:**
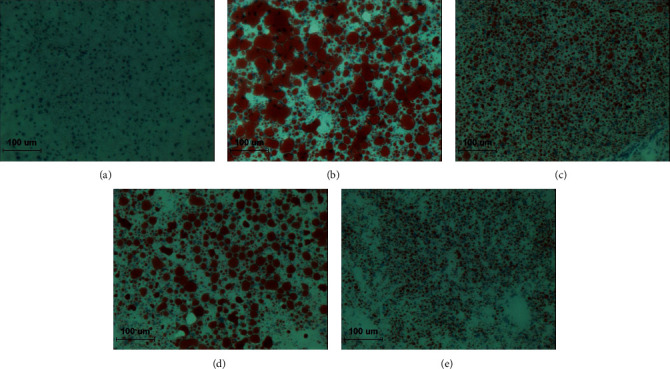
Oil red O staining of hepatic tissue. (a) Normal: normal mice; (b) Control: HFD control mice; (c) GCE200: GCE 200 mg/kg-treated and obese mice; (d) TCN100: TCN 100 mg/kg-treated and obese mice; (e) TCN200: TCN 200 mg/kg-treated and obese mice. ×200.

As shown in Figure 5, we investigate whether the improved lipid metabolism had changed at the lipid accumulation. We observed Oil red O staining in the liver to evaluate the extent of lipid accumulation. As expected, mice fed only HFD resulted in severe hepatic lipid accumulation, while GCE and TCN administration more effectively improved the pathological condition compared with the HFD control group.

### 3.6. LKB1/AMPK/ACC Phosphorylation Protein Expressions in the Liver

**Figure 6:**
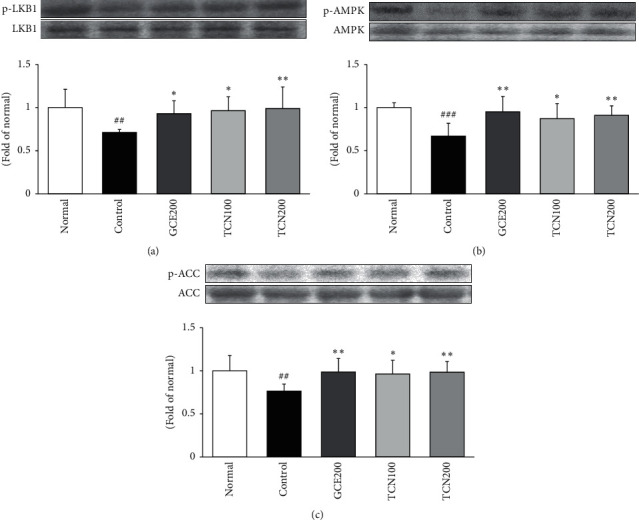
LKB1/AMPK/p-ACC expressions in the liver of mice fed HFD. Western blot analysis of LKB1, phosphorylated LKB1 (p-LKB1), AMPK, phosphorylated AMPK (p-AMPK), phosphorylated ACC (p-ACC), and ACC proteins. Normal: normal mice; Control: HFD control mice; GCE200: GCE 200 mg/kg-treated and obese mice; TCN100: TCN 100 mg/kg-treated and obese mice; TCN200: TCN 200 mg/kg-treated and obese mice. Data are the mean ± SD, (*n* = 7). Significance: ^*∗*^*p* < 0.05, ^*∗∗*^*p* < 0.01 versus the HFD control mice. The blots shown are representative of three blots from each group of mice.

As shown in Figure 6, p-LKB1/p-AMPK/p-ACC protein levels were significantly decreased in the group fed only HFD compared with the normal group, while the reduced protein levels were significantly increased in both GCE and TCN treatment.

### 3.7. *β*-Oxidation-Related Protein Expressions in the Liver

**Figure 7:**
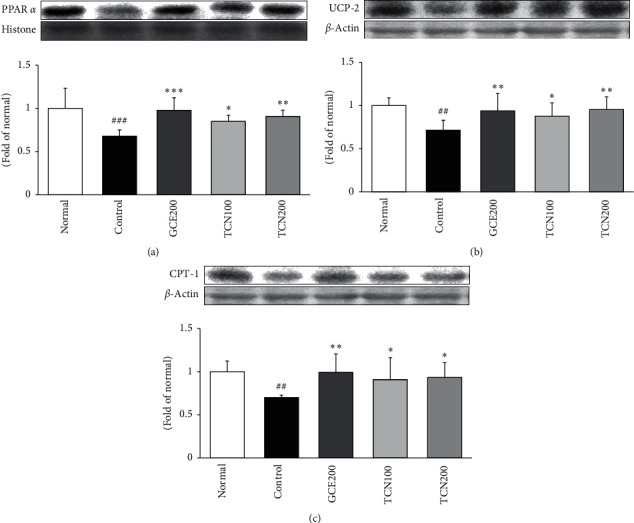
PPAR*α*, UCP-2, and CPT-1 expressions in the liver of mice fed HFD. Western blot analysis of PPAR*α*, UCP-2, and CPT-1 proteins. Normal: normal mice; Control: HFD control mice; GCE200: GCE 200 mg/kg-treated and obese mice; TCN100: TCN 100 mg/kg-treated and obese mice; TCN200: TCN 200 mg/kg-treated and obese mice. Data are the mean ± SD (*n* = 7). Significance: ^*∗*^*p* < 0.05, ^*∗∗*^*p* < 0.01 versus the HFD control mice. The blots shown are representative of three blots from each group of mice. PPAR*α*: peroxisome proliferator activated receptor *α*; UCP-2: uncoupling protein 2; CPT-1: carnitine palmitoyltransferase 1A.

Figure 7 reveals that mice fed HFD remarkably reduced the protein expressions of *β*-oxidation markers such as PPAR*α*, CPT-1, and UCP-2. However, TCN treatment significantly reversed the decreased PPAR*α* expression. Moreover, its target gene, CPT-1 and UCP-2 by TCN supplementation significantly increased compared with those of the HFD control group.

## 4. Discussion

Obesity as a growing worldwide concern is determined by abnormal fat deposition, which may have negative effects on general health status. Accordingly, the management of related risk factors like dyslipidemia, hypertension, cardiovascular diseases, type 2 diabetes, and metabolic syndrome through body weight loss is an important concern in obesity control [25]. The various therapeutic approaches such as lifestyle changes, medications, and surgery are introduced for obesity treatment. Despite acquiring partially desirable results, the problem remained unsolved. Therefore, a new approach like the use of safe and effective natural herb seems to be a promising and novel strategy to control obesity and related syndromes because of being able to overcome previous limitations [26].

This work demonstrated for the first attempt the effect and the underlying mechanism of TCN treatment on lipid metabolism and *β*-oxidation. First of all, DPPH and ABTS radical scavenging activities were performed to compare antioxidant activities according to parts (leaf and root) and extraction methods (water, 30% ethanol, and 70% ethanol) of TCN for the selection of sample to be used in animal testing. Antioxidant activity is expressed in terms of IC_50,_ and IC_50_ (*μ*g/mL) represents half maximal inhibitory concentration of samples to scavenge DPPH and ABTS radical. As shown in Figure 2, IC_50_ values of DPPH radical scavenging activity of leaf and root extracted in 70% ethanol extract were 164.06 ± 2.51 and 166.51 ± 6.3 *μ*g/mL. Also, IC_50_ value of ABTS radical scavenging activity was 257.12 ± 3.77 and 162.7 ± 0.6 *μ*g/mL. The 70% ethanol extract showed better radical scavenging than that of water extract. Subsequently, assays for the content measurement of the phenolic compound or flavonoid were performed. Total phenolic content of 70% ethanol extract (leaf) was the best among the six samples as 25.74 ± 0.17 mg GAE/g of TCN extract. The flavonoid content also had the highest content as 12.53 ± 0.03 mg naringin equivalent (NE)/g of TCN extract. Taken together, 70% ethanol extract (leaf) was most excellent in all antioxidant capabilities. The ex vivo lipolysis assay was performed additionally to compare lipolysis effect according to parts (leaf and root) and extraction methods (water, 30% ethanol, and 70% ethanol) of TCN. As shown in Figure 3, the amount of glycerol spilled of leaf extracted using 70% ethanol extract was 74.63 ± 0.59% (fold of isoproterenol). Leaf sample extracted using 70% ethanol extract among 6 sample extracts was the most outstanding sample in lipolysis effect. As a result, the sample used in this experiment was determined to be 70% ethanol extract (leaf).

Herein, we showed that *Taraxacum coreanum* Nakai (TCN) functioned its antiobesity effect on HFD induced obese mice model and then affected two processes: (1) increased LKB1/AMPK signaling pathway which resulted in the phosphorylation of ACC. P-AMPK activated by LKB1 (AMPK kinases) works to enhance *β*-oxidation by the phosphorylation of acetyl-CoA carboxylase (ACC), thereby increasing fatty acid degradation. (2) activated expression of PPAR*α*, which is an important regulator of peroxisomal and mitochondrial *β*-oxidation of fatty acids and lipid metabolism [27].

Table 2 shows body weight gain, food intake, and food efficiency ratio (FER) during the experimental periods. Mean body weights of all groups started without a significant difference between 22.27 g and 22.99 g. Normal mice increased slightly in weight gain during the experimental period of 5 weeks and the level showed a significant difference compared with the HFD control group (*p* < 0.001). GCE200 and TCN200 treated groups were significantly decreased compared with the HFD control group (GCE200; *p* < 0.001, TCN200; *p* < 0.01). Moreover, the food intake and food efficiency ratio among HFD supplied groups did not show a significant difference. As a result, TCN treatment decreased body weight gain without a significant change of FER. Furthermore, we revealed that TCN-treated HFD mice displayed an outstanding decrease in body weight and improved dyslipidemia; this effect was similar to that of *Garcinia cambogia.* HFD caused the increase in serum TG, TC, HDL-cholesterol, LDL-cholesterol, and VLDL-cholesterol (*p* < 0.001, respectively). The elevated TG and VLDL-cholesterol levels were significantly reduced by all drugs compared with the HFD control mice (*p* < 0.001). Moreover, the augmented TC and LDL-cholesterol levels were significantly lowered by GCE and TCN compared with the HFD control mice (GCE200; *p* < 0.01, TCN100; *p* < 0.05, TCN200; *p* < 0.01), whereas the administration of GCE and TCN significantly elevated HDL-cholesterol level (*p* < 0.001). Particularly, TG and VLDL-cholesterol in TCN200 group showed lower values than those of normal group (Figure 4). TCN treatment significantly reduced serum TG, TC, LDL-cholesterol, and VLDL-cholesterol levels, while it significantly enhanced HDL-cholesterol. In addition, the histological alterations of the liver by Oil Red O staining showed that TCN supplementation obviously reversed the increased lipid accumulation in the liver (Figure 5).

Accumulating evidence of various studies has shown that AMPK plays core roles in regulating lipid metabolism in the liver. Herein, AMPK activation promotes *β*-oxidation and downregulates fatty acid biosynthesis [28]. Recent studies have reported that AMPK occurs with the phosphorylation of the *α*-subunit at threonine 172 (Thr172) by upstream kinases (LKB1) because of increase of cellular AMP : ATP ratio. The phosphorlyation of ACC results in decreased malonyl CoA levels. Thereby, a fall in malonyl CoA disinhibits CPT-1, which is a rate-limiting step for the entry into the mitochondria for *β*-oxidation [29]. This series of pathway is the central effect of AMPK to decrease lipid stores in the liver. The previous reports showed that AMPK phosphorylation levels reduced in the liver after HFD intake [30]. In the current study, we found that p-LKB1/p-AMPK/p-ACC protein levels were significantly decreased in the group fed only HFD compared with the normal group, while the reduced protein levels were significantly increased by TCN treatment (Figure 6). These will increase energy production such as a reduction of energy utilization and an increase of *β*-oxidation.

Peroxisome proliferator-activated receptors (PPARs) have three different isoforms such as -*α*, -*β*, and -*γ* [31]. The previous studies proposed that PPAR*α* mediated transcription is shown to be co-activated by AMPK [32]. PPAR*α* is involved in fatty acid catabolism including *β*-oxidation pathway and is most abundant in the liver and adipose tissue [33] activation of the expression of genes associated with the *β*-oxidation pathway [34]. PPAR*α* expression was positively correlated with the expression of CPT-1. Namely, PPAR*α* regulates the levels of CPT-1, an enzyme essential for *β*-oxidation of long-chain fatty acids [35, 36]. The present study indicated a similar tendency of PPAR*α* and CPT-1 expressions [37, 38]. Figure 7 reveals that mice fed HFD remarkably reduced the protein expressions of *β*-oxidation markers such as PPAR*α* and CPT-1. However, TCN treatment significantly reversed the decreased expressions. Furthermore, many studies have reported that PPAR*α* activators upregulate mitochondrial uncoupling protein- (UCP-) 2 expression in the liver. PPAR*α* works to accelerate the thermogenic protein such as UCP-2 as a means for limiting the production of ROS and regulating lipid metabolism [28, 39]. Our result also showed that UCP-2 was significantly upregulated by TCN treatment via PPAR*α* activation.

Besides, several inflammatory markers have been associated with both obesity and risk of adverse outcomes in obesity-associated diseases [40]. Accordingly, the mechanism study underpinning the triggers of such inflammatory responses after TCN treatment could offer another strategy to ameliorate the risk of obesity-associated disease.

## 5. Conclusions

**Figure 8:**
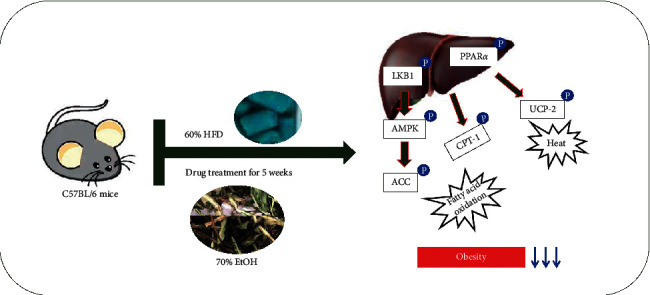
Predicted mechanism after administering TCN in the liver.

In this work, we studied the antiobesity effect with the 70% ethanol root extract selected by comparing and analyzing the effects of the extraction method of the leaves and roots of *Taraxacum coreanum* Nakai. Taken together, the current study suggests that TCN supplementation regulates lipid metabolism via LKB1-AMPK signaling pathway and promotes *β*-oxidation by PPAR*α*, as shown in Figure 8. Hence, TCN may have potential remedy in the prevention and treatment of obesity. However, it is judged that additional studies are needed to determine which effective components of TCN exhibited antiobesity effect.

## Figures and Tables

**Table 1:** Extract yields according to different extraction methods of leaf and root of TCN.

Extract methods	Leaf (%)	Root (%)
Water extract	15.5	14.5
30% ethanol extract	19.3	17.0
70% ethanol extract	21.5	11.1

**Table 2:** Body weight gain, liver weight, and food efficiency ratio.

Group	Body weight			Liver weight	Food intake	Food efficiency ratio
	Initial (g)	Final (g)	Gain (g/day)	(mg/g B.W)	(g/day)	(%)
Normal	22.27 ± 0.26	26.16 ± 0.35	0.11 ± 0.01	30.99 ± 1.71	2.56 ± 0.10	4.47 ± 0.67
HFD-fed mice						
Control	22.92 ± 0.18	32.23 ± 0.75^###^	0.27 ± 0.01^###^	37.86 ± 3.98^##^	1.91 ± 0.03^###^	12.53 ± 0.97^###^
GCE200	22.99 ± 0.23	29.09 ± 0.83^*∗∗∗*^	0.19 ± 0.02^*∗∗∗*^	26.12 ± 4.60^*∗∗∗*^	1.94 ± 0.11	10.93 ± 2.99
TCN100	22.94 ± 0.30	30.87 ± 1.08	0.24 ± 1.02	30.83 ± 5.10^*∗∗*^	1.99 ± 0.07	12.34 ± 4.39
TCN200	22.91 ± 0.24	29.77 ± 0.62^*∗∗*^	0.20 ± 0.01^*∗∗*^	27.79 ± 8.87^*∗∗∗*^	1.94 ± 0.06	11.58 ± 3.06

Control: HFD control mice; GCE: GCE 200 mg/kg-treated and HFD-fed mice; TCN100: TCN 100 mg/kg-treated and HFD-fed mice; TCN200: TCN 200 mg/kg-treated and HFD-fed mice. Data are the mean ± SD (*n* = 8). Significance: ^###^*p* < 0.001 versus the normal mice and ^*∗∗*^*p* < 0.01, ^*∗∗∗*^*p* < 0.001 versus the HFD control mice.

## Data Availability

The datasets used and analyzed in this work are available from the corresponding author upon reasonable request.

## References

[1] Arroyo-Johnson C., Mincey K. D. (2016). Obesity epidemiology worldwide. *Gastroenterology Clinics of North America*.

[2] Pereira K., Salsamendi J., Casillas J. (2015). The global nonalcoholic fatty liver disease epidemic: what a radiologist needs to know. *Journal of Clinical Imaging Science*.

[3] Ahirwar R., Mondal P. R. (2019). Prevalence of obesity in India: a systematic review. *Diabetes & Metabolic Syndrome: Clinical Research & Reviews*.

[4] Saltiel A. R., Olefsky J. M. (2017). Inflammatory mechanisms linking obesity and metabolic disease. *Journal of Clinical Investigation*.

[5] Jeon S.-M. (2016). Regulation and function of AMPK in physiology and diseases. *Experimental & Molecular Medicine*.

[6] Yan Y., Zhou X., Xu H., Melcher K. (2018). Structure and physiological regulation of AMPK. *International Journal of Molecular Sciences*.

[7] Gómez-Galeno J. E., Dang Q., Nguyen T. H. (2010). A potent and selective AMPK activator that inhibits de novo lipogenesis. *ACS Medicinal Chemistry Letters*.

[8] Momtaz S., Salek-Maghsoudi A., Abdolghaffari A. H. (2019). Polyphenols targeting diabetes via the AMP-activated protein kinase pathway; future approach to drug discovery. *Critical Reviews in Clinical Laboratory Sciences*.

[9] Yoneda M., Guo Y., Ono H. (2010). Decreased SIRT1 expression and LKB1 phosphorylation occur with long-term high-fat diet feeding, in addition to AMPK phosphorylation impairment in the early phase. *Obesity Research & Clinical Practice*.

[10] Yang H. J., Kim M. J., Kwon D. Y., Kang E. S., Kang S., Park S. (2017). Gastroprotective actions of Taraxacum coreanum Nakai water extracts in ethanol-induced rat models of acute and chronic gastritis. *Journal of Ethnopharmacology*.

[11] Yoon C.-S., Ko W., Lee D.-S. (2017). Taraxacum coreanum protects against glutamate-induced neurotoxicity through heme oxygenase-1 expression in mouse hippocampal HT22 cells. *Molecular Medicine Reports*.

[12] Gulfraz M., Ahamd D., Ahmad M. S. (2014). Effect of leaf extracts of Taraxacum officinale on CCl_4_ induced hepatotoxicity in rats, in vivo study. *Pakistan Journal of Pharmaceutical Sciences*.

[13] Park S., Kim D. S., Wu X., Yi Q. J. (2018). Mulberry and dandelion water extracts prevent alcohol-induced steatosis with alleviating gut microbiome dysbiosis. *Experimental Biology and Medicine*.

[14] Hfaiedh M., Brahmi D., Zourgui L. (2016). Hepatoprotective effect of Taraxacum officinale leaf extract on sodium dichromate-induced liver injury in rats. *Environmental Toxicology*.

[15] Schütz K., Carle R., Schieber A. (2016). Taraxacum—a review on its phytochemical and pharmacological profile. *Journal of Ethnopharmacology*.

[16] Lee K., Whang W. (2018). Inhibitory effects of bioassay-guided isolation of anti-glycation components from Taraxacum coreanum and simultaneous quantification. *Molecules*.

[17] Hatano T., Edamatsu R., Hiramatsu M. (1989). Effects of the interaction of tannins with co-existing substances. VI. Effects of tannins and related polyphenols on superoxide anion radical, and on 1,1-diphenyl-2-picrylhydrazyl radical. *Chemical & Pharmaceutical Bulletin*.

[18] Re R., Pellegrini N., Proteggente A. (1999). Antioxidant activity applying an improved ABTS radical cation decolorization assay. *Free Radical Biology and Medicine*.

[19] Slinkard K., Singleton V. L. (1997). Total phenol analysis: automation and comparison with manual methods. *American Journal of Enology and Viticulture*.

[20] Lister C. E., Lancaster J. E., Sutton K. H., Walker J. R. L. (1994). Developmental changes in the concentration and composition of flavonoids in skin of a red and a green apple cultivar. *Journal of the Science of Food and Agriculture*.

[21] Hu C. (2018). Taraxacum: phytochemistry and health benefits. *Chinese Herbal Medicines*.

[22] Komatsu S. (2007). Extraction of nuclear proteins. *Methods in Molecular Biology*.

[23] Shin M. R., Lee J. A., Kim M. J. (2020). Protective effects of phellinus linteus mycelium on the development of osteoarthritis after monosodium iodoacetate injection. *Evidence-Based Complementary and Alternative Medicine*.

[24] Kim S. H., Shin M. R., Lee A. R., Seo B. I., Park H. J., Roh S. S. (2020). Improvement of inflammation through antioxidant pathway of gardeniae fructus 50% EtOH extract (GE) from acute reflux esophagitis rats. *BioMed Research International*.

[25] Payab M., Goodarzi P., Foroughi Heravani N. (2018). Stem cell and obesity: current state and future perspective. *Advances in Experimental Medicine and Biology*.

[26] Hu J., Li X., Tian W. (2019). Adenanthin, a natural ent-kaurane diterpenoid isolated from the herb isodon adenantha inhibits adipogenesis and the development of obesity by regulation of ROS. *Molecules*.

[27] Xie Z., Zhang J., Wu J., Viollet B., Zou M.-H. (2008). Upregulation of mitochondrial uncoupling protein-2 by the AMP-activated protein kinase in endothelial cells attenuates oxidative stress in diabetes. *Diabetes*.

[28] Kersten S. (2014). Integrated physiology and systems biology of PPAR*α*. *Molecular Metabolism*.

[29] Seo M. S., Kim J. H., Kim H. J., Chang K. C., Park S. W. (2015). Honokiol activates the LKB1-AMPK signaling pathway and attenuates the lipid accumulation in hepatocytes. *Toxicology and Applied Pharmacology*.

[30] Kahn B. B., Alquier T., Carling D., Hardie D. G. (2015). AMP-activated protein kinase: ancient energy gauge provides clues to modern understanding of metabolism. *Cell Metabolism*.

[31] Ha S.-K., Kim J., Chae C. (2011). Role of AMP-activated protein kinase and adiponectin during development of hepatic steatosis in high-fat diet-induced obesity in rats. *Journal of Comparative Pathology*.

[32] Debril M.-B., Renaud J.-P., Fajas L., Auwerx J. (2001). The pleiotropic functions of peroxisome proliferator-activated receptor *γ*. *Journal of Molecular Medicine*.

[33] Bronner M., Hertz R., Bar-Tana J. (2004). Kinase-independent transcriptional co-activation of peroxisome proliferator-activated receptor alpha by AMP-activated protein kinase. *The Biochemical Journal*.

[34] Chinetti G., Fruchart J.-C., Staels B. (2000). Peroxisome proliferator-activated receptors (PPARs): nuclear receptors at the crossroads between lipid metabolism and inflammation. *Inflammation Research*.

[35] Ohashi T., Nakade Y., Ibusuki M. (2019). Conophylline inhibits high fat diet-induced non-alcoholic fatty liver disease in mice. *PLoS One*.

[36] Nagy T. R., Blaylock M. L., Garvey W. T. (2004). Role of UCP2 and UCP3 in nutrition and obesity. *Nutrition*.

[37] Hsu S.-C., Huang C.-j. (2007). Changes in liver PPAR*α* mRNA expression in response to two levels of high-safflower-oil diets correlate with changes in adiposity and serum leptin in rats and mice. *The Journal of Nutritional Biochemistry*.

[38] Lei L., Xiaoyi S., Fuchang L. (2017). Effect of dietary copper addition on lipid metabolism in rabbits. *Food & Nutrition Research*.

[39] Ma S., Yang D., Li D., Tan Y., Tang B., Yang Y. (2012). Inhibition of uncoupling protein 2 with genipin exacerbates palmitate-induced hepatic steatosis. *Lipids in Health and Disease*.

[40] Cox A. J., West N. P., Cripps A. W. (2015). Obesity, inflammation, and the gut microbiota. *The Lancet Diabetes & Endocrinology*.

